# Synthesis and Ready to Use Kit Formulation of EDTMP for the Preparation of 177Lu-EDTMP as a Bone Palliation Radiopharmaceutical

**DOI:** 10.2174/1874471015666220721095938

**Published:** 2023-01-31

**Authors:** Guldem Mercanoglu, Kani Zilbeyaz, Nuri Arslan

**Affiliations:** 1 Department of Pharmacology, Hamidiye Pharmacy Faculty, University of Health Sciences, Istanbul, Turkey;; 2 Department of Chemistry, Faculty of Art and Sciences, Ağrı İbrahim Çeçen University, Ağrı, Turkey;; 3 Department of Nuclear Medicine, Faculty of Medicine, Near East University, Lefkoşa- Cyprus

**Keywords:** Bone palliation, EDTMP, ^177^Lu-EDTMP, ready-to-use kit, radiopharmaceuticals

## Abstract

**Introduction:**

With its suitable nuclear decay characteristics and large-scale production feasibility with adequate specific activity, ^177^Lu is regarded as an excellent radionuclide for developing bone pain palliation agent. Ethylenediamine-tetramethylene phosphonic acid (EDTMP) is a preferred carrier molecule for radiolanthanides, such as ^177^Lu. The present paper describes the synthesis of EDTMP and the development of a ready-to-use kit for the preparation of ^177^Lu-EDTMP and its quality control in accordance with the quality and safety criteria required for medicinal use.

**Material and Methods:**

EDTMP was synthesized by a modified Mannich-type reaction, and the structure was characterized using NMR and IR spectroscopy. Optimization of radiolabeling conditions was done with two different salt forms of EDTMP. The labeling yield was checked by paper chromatography with radiation detection. Kit was developed as a lyophilized mixture of EDTMP and sodium bicarbonate in a maximum volume of 5 mL. Labeling efficiency, radionuclidic purity, radiochemical purity, sterility, and pyrogenicity analysis were performed as the quality control of the labeled kit.

**Results:**

The analytical data for the structure determination and purity of the synthesized ligand were in agreement with authentic commercial samples used in radiopharmacy.^177^Lu-EDTMP complex was prepared using synthesized EDTMP ligand under optimized labeling conditions with high labelling yield (>99%). The radiolabeling yields of the EDTMP kit at room temperature after 30 min and 48 hours were 99.46% and 99.00%.

**Conclusion:**

The developed EDTMP kit enables an instant one-step preparation of the radiopharmaceutical of high radiochemical purity (>99%) and has a sufficiently long shelf life. This enables the routine production of the ^177^Lu-EDTMP in nuclear medicine clinics without requiring experienced staff.

## 1 INTRODUCTION

In the advanced stages of the disease, a large percentage of patients with primary carcinoma of the breast, lung, or prostate develop metastasis in their bones, resulting in excruciating pain, hypercalcemia, and a lack of mobility [1]. Systematic palliative therapy with radiopharmaceuticals has been reported to be a very effective treatment modality for patients with multiple skeletal lesions compared to other conventional methods, such as analgesics and external beam radiotherapy [2, 3]. Radiopharmaceuticals are radioactive drugs that are used to diagnose and treat a wide range of pathological conditions. The key mechanism behind their effects is targeting diseases using specific carrier molecules labeled with radionuclides. The major challenge in the development of an effective radiopharmaceutical for bone palliation is delivering an adequate dose of radiation at the lesion with minimum radiation-induced bone marrow suppression [4, 5]. This is determined by the energy and the penetrating ability of the particulates emitted from the radionuclide part of the radiopharmaceutical [6, 7]. With ^177^Lu, a β^-^emitter, minimal bone marrow suppression can be predicted while delivering the appropriate dose to skeletal lesions [8]. Again, gamma photon emission with low abundance and sufficient energy from ^177^Lu allows for simultaneous scintigraphy studies and dosimetric evaluation [9]. Due to its high bone uptake, selective localization in skeletal lesions, and ability to form metal chelates with high *in-vivo* stability, especially with lanthanides, ethylenediamine tetramethylene phosphonic acid (EDTMP), a multidentate polyaminophosphonic acid, is a widely used carrier ligand [10].

Radiopharmaceuticals can be prepared using ready-to-use kits or automated synthesis modules in hospital radiopharmacy laboratories. The second way requires specialized personnel and armored areas (hot-lab) for both radioprotection and good manufacturing practices (GMP) requirements. The high costs in creating relevant areas and the shortage of expert personnel (radiopharmacists) are the leading operational challenges for preparing radiopharmaceuticals by using automated synthesis modules. Compared to these challenges, the preparation of radiopharmaceuticals by using ready-to-use kits (shake and bake kits) allows radiopharmaceuticals to be administrated to the patient after a simple reconstitution process in Nuclear Medicine clinics. The availability of freeze-dried cold kits plays an essential role in the extensive use of ^99m^Tc radiopharmaceuticals [11]. Cold kits are also suitable for the preparation of radiopharmaceuticals, labelled with either generator-produced radionuclides or long-lived radionuclides produced in nuclear reactors [11]. Therapeutic radiopharmaceuticals, such as the ^177^Lu labeled radiopharmaceuticals with a relatively long half-life, can also be prepared using cold kits of the ligands. An example is ready to use kit of prostate-specific membrane antigen ligand (PSMA-11) for the preparation of the ^177^Lu-PSMA11. Although there are many studies in the literature on both the labeling of EDTMP with ^177^Lu and the use of ^177^Lu-EDTMP in the palliative treatment of bone metastases [12-14], all of these studies use different labeling procedures. This study aims to develop a ready-to-use kit formulation to prepare ^177^Lu-EDTMP for use in routine nuclear medicine applications.

## 2 MATERIALS AND METHODS

^177^LuCl_3_ was obtained from Polatom (Otwock, Poland) with a specific activity of around 750 GBq/mg. All the chemicals used for the synthesis of the ligands were purchased from Aldrich Chemical Company, USA. All other chemicals were supplied from a local manufacturer (InterLab, Turkey). Whatman 3MM chromatography paper (UK) was used for paper chromatography studies. EDTMP was synthesized in-house by the method reported by Mehri and co-workers [15] and characterized by infrared (FT-IR - Perkin-Elmer, USA) and nuclear magnetic resonance spectroscopy (Bruker spectrometer - Bruker, USA).

Radioactivity measurements were made using a NaI (Tl) scintillation counter on adjustment of the baseline at 150 keV and keeping a window of 100 keV for utilizing the 208 keV (11%) g-photopeak of ^177^Lu for detection.

Labeling efficiency and radionuclidic purity were determined by paper chromatography with radiation detection (Miniscan Radio-TLC Scanner, Eckert & Ziegler, Germany), and radiochemical purity was determined by radio-HPLC (Modular-Lab HPLC, Eckert & Ziegler, Germany).

### 2.1 Synthesis of EDTMP

EDTMP was synthesized from phosphorous acid, ethylenediamine, and formaldehyde in the presence of HCl by a modified Mannich-type reaction [15]. To a mixture of water and acid (100mL of water and 50 mL of 37% HCl) in a round bottom flask, 0.1 mol of ethylamine and 0.4 mole of phosphorous acid were added. The mixture was stirred to dissolve the contents and then refluxed for 2 hours. To the reaction, formaldehyde (37%, 0.4 mol) was added dropwise, and the resulting mixture was refluxed for a further 4 hours. After this period, the obtained solution was cooled at room temperature. Subsequently, the solvent was removed under reduced pressure using a rotary evaporator. Ethanol was added to the concentrated solution, which produced a white precipitate. The product was obtained by filtration, washed with ethanol, and then dried under vacuum. The synthesis scheme is shown in Fig. (**1**).

The structure was characterized using ^1^H-NMR, ^13^C-NMR, and IR spectroscopy.

#### 2.1.1 ^1^H and ^13^C NMR Spectroscopy

Samples were dissolved in D_2_O. Chemical shifts (δ) are given in ppm, and coupling constants (J) are given in Hz.

^1^H-NMR (D_2_O): δ= 3.12 (d, J= 11.2 Hz, 8H, 4 x CH2-P), 3.40 (s, 4H, N- CH_2_-CH2-N)

^13^C-NMR (D_2_O, ppm): δ= 52.2 (CH2-N), 53.5 (CH2-P)

#### 2.1.2 IR Spectroscopy

FT-IR spectrum was obtained using the KBr pellet technique at the interval of 500-4000 cm^-1^ in the solid phase of the sample. IR spectra of the samples recorded with FT-IR spectrometer are given as wave numbers:

IR (KBr, ν cm^-1^): 3419.5 (OH); 2610.6 (PO-H); 1668 (O=P-O-H); 1436.8 (C-N); 1262.2 (P=O)

### 2.2 Optimization of ^177^Lu-EDTMP Labeling Conditions

The optimization study was carried out with two different salt forms of EDTMP according to our previous labeling study with some modifications [16]. Each labelling was done in triplicate.

#### 2.2.1 Natrium Salt of EDTMP

A total of 35 mg of EDTMP was dissolved in an aqueous solution of NaHCO_3_ (0.5 M, 0.5 mL), and the pH was adjusted to 8.0. The prepared solution was incubated with ^177^LuCl_3_ (100 mCi, 0.2 mL) for 30 minutes, and the final volume was adjusted to 5 ml with isotonic sodium chloride.

#### 2.2.2 Natrium-Calcium Salt of EDTMP

A total of 5.72 mg of CaCO_3_ (0.143mM) was dispersed in 0.4 mL of purified water. A total of 35 mg of EDTMP was added to the dispersion and stirred. The pH was adjusted to 7.0 with aqueous solution of NaOH (1M). The prepared solution was incubated with ^177^LuCl_3_ (100 mCi, 0.2 mL) for 30 minutes, and the final volume was adjusted to 5 ml with isotonic sodium chloride.

#### 2.2.3 Determination of Radiolabeling Yields

Radiolabeling yields were determined by paper chromatography with radiation detection. Two ⎧l of the test sample was applied at 1.0 cm from the bottom end of the paper strip (10x2 cm), and the strip was developed in NH_4_OH: H_2_O: CH_3_OH (2.5:50:50) for 8 cm and dried. The dried strips were placed on the scanner coupled witha Na(I) detector and scanned to get chromatograms.

### 2.3 Formulation of Kit and Quality Control

Similar radiolabeling yields were determined for both salt forms of EDTMP in the optimization studies, and thus the kit formulation study was carried out with the sodium salt of EDTMP. Accordingly, 35 mg of EDTMP was dissolved in 0.5 ml of 0.5M NaHCO_3_, pH was adjusted to 8.0, and the final volume was made up to 5 ml with isotonic sodium chloride. The prepared solution was filtrated through a 0.22- ⎧m membrane filter, dispensed into a 10-ml vial and lyophilized. Lyophilization was performed for 24 h with a shelf temperature of -80°C and 0.630 mbar pressure. Vials were capped under vacuum and stored between 2-8 ºC. The kit content was incubated with 100mCi ^177^LuCl_3_ solution (0.2 mL in HCl) at room temperature for 30 minutes with continuous mixing. After controlling the pH, the final volume was adjusted to 5 ml with isotonic sodium chloride. Labeling efficiency, radionuclidic purity, radiochemical purity, sterility, and pyrogenic contamination analysis were performed as the quality control of the ^177^Lu-EDTMP. The labeling efficiency and radionuclidic purity were determined by paper chromatography, as described in section 2.2.3

Radiochemical purity was assessed by radio-HPLC. A total of 20 μl of the test solution was injected into the C‐18 reversed phase (25 × 0.5 cm) column, and the elution was monitored. Chromatograms were obtained on an HPLC system with a Na(I) crystal detector, using a gradient mobile phase system 100% A (H_2_O: C_2_H_5_OH (90:10) for 0-5 min), 100% B (H_2_O:C_2_HF_3_O_2_ (100:0.1) for 5-20 min).

As for the microbial quality control tests, the sterility was performed by direct inoculation technique, and the pyrogenic contaminations were assessed by the Limulus amebocyte lysate (LAL) test.

### 2.4 Stability Studies

The stability of ^177^Lu-EDTMP was studied at room temperature by determining the radiochemical purity of the complex at 0, 6, 12, 24, and 48 hours by radio HPLC, as described in Section 2.3.

## 3 RESULTS

EDTMP ligand was synthesized in-house, and the structure was characterized using ^1^H-NMR, ^13^C-NMR, and IR spectroscopy. The results obtained were in agreement with authentic commercial samples used in radiopharmacy.

### 3.1 Optimization of Radiolabeling and Determination of Labeling Yield

**Fig. (2):**
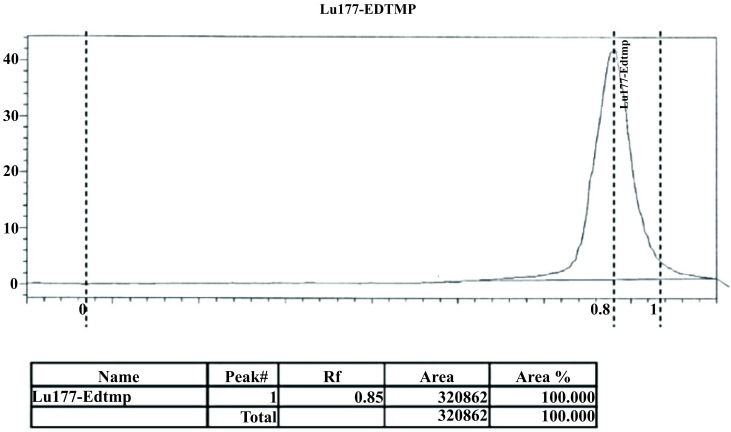
Sample chromatogram of ^177^Lu-EDTMP complex determined by Na(I) detector in paper chromatography.

Radiolabeling optimization studies were conducted with two different salts of EDTMP. Characterization of the ^177^Lu-EDTMP complexes and the determination of the radiolabeling yields were assessed by paper chromatography with radiation detection. For the paper chromatography, NH_4_OH: H_2_O: CH_3_ OH (2.5:50:50) was used as the eluting solvent. The ^177^Lu-EDTMP complexes moved towards the solvent front (R_f_ = 0.85) while uncomplexed ^177^LuCl_3_ remained at the spotting point (R_f_ = 0.1) (Fig. **2**).

Labeling yields were expressed as the percentage of total activity counted by the Na(I) detector. Both natrium and natrium/calcium salts of the ^177^Lu-EDTMP complexes were obtained in very high yields (> 98%) Table **1**. Similar radiolabeling yields were obtained for both salts of EDTMP, and thus the kit formulation study was conducted with the sodium salt of EDTMP in consideration of the ease of preparation.

### 3.2 Formulation of the Kit and Quality Control

Each kit vial contains a lyophilized mixture of 35 mg EDTMP, 21 mg NaHCO_3_ in a maximum volume of 5 mL and is capable of producing up to 3.7 GBq (100 mCi) patient dose of ^177^Lu-EDTMP with the standard procedure (*i.e*., 30 min incubation with continuous mixing at room temperature). Labeling efficiency, radionuclidic purity, radiochemical purity, sterility, and pyrogenicity analysis were performed as the quality control of the labeled kit.

**Fig. (3):**
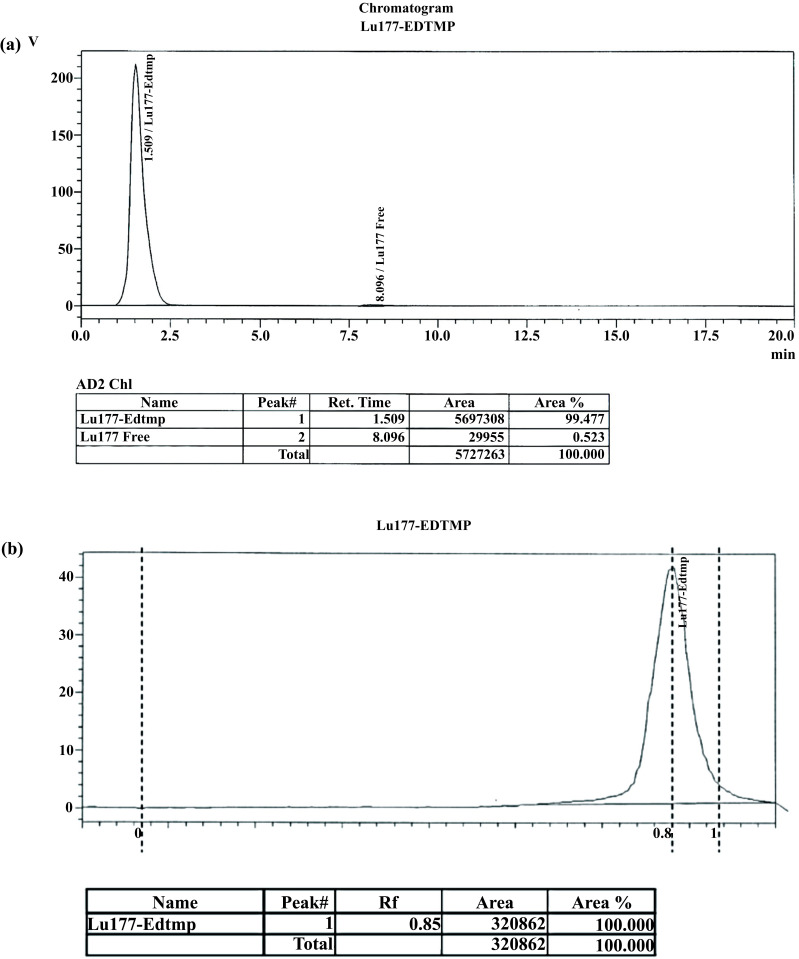
(**a**), Radionuclidic and radiochemical purities of 177Lu-labeled kits determined by radio-HPLC (**b**), and Paper chromatography.

After elution, the dried paper strips were scanned for the evaluation of the radionuclidic purity and the labeling efficiency. The scanner generated radio chromatograms that showed 98% labeling of ^177^Lu-EDTMP. The complex moved towards the solvent front while free lutetium remained at the origin. The chromatogram confirmed a peak at Rf=0.85, indicating ^177^Lu-EDTMP and a peak at Rf=0.0 for ^177^LuCl_3_ (Fig. **3**).

Regarding the radiochemical purity, the HPLC chromatogram of the sample solution clearly showed a distinct peak, confirming the labeling of ^177^Lu with EDTMP. As seen in Fig. (**3**), it was observed that the retention time of ^177^Lu-EDTMP was 1.5 min, while that of free ^177^LuCl3 was found to be 8.0 min. HPLC results were in close agreement with those of paper chromatography.

The microbial quality of the kits was guaranteed by the sterility and the sterile and pyrogen-free ^177^Lu complex guaranteed the microbial quality of the kits of the ^177^Lu- complex. This indicates that the ligand labeled with ^177^Lu can be administrated parenterally.

### 3.3 Stability of 1^77^Lu-EDTMP

The stability of the prepared ^177^Lu-EDTMP complex was studied up to 48 h after preparation. High radiochemical purity was found in ^177^Lu-EDTMP preparation during storage at room temperature. A radiochemical purity >98% was observed over the period of 48h after labeling by using Whatman 3 MM eluted with NH_4_OH: H_2_O: CH_3_OH (2.5:50:50) mixture (Table **2**). This is due to a high specific activity of ^177^Lu (around 750 GBq/mg), which results in an increased ligand-to-metal ratio in the complex [17].

## 4 DISCUSSION

In daily nuclear medicine practices, the use of kit-based radiopharmaceuticals has some advantages: (1) long shelf life with convenient transportation conditions; (2) procedural reliability; (3) high radionuclidic and radiochemical purity by labeling of the kit contents with the radionuclide just before the use; (4) assured sterility; (5) ease of preparation with minimum clean room conditions and experienced personnel; (6) no need for final purification [18]. Therefore, using ready-to-use kits in hospital settings can help to produce high-quality, repeatable, and standardized radiopharmaceuticals with minimum time, cost, and experienced personnel.

The large-scale production of ^177^Lu with excellent radionuclide purity and adequate specific activity makes it a suitable candidate for therapeutic applications. Currently, much interest is being shown in the use of ^177^Lu for various applications in nuclear medicine practices. Although various agents labeled with ^32^P, ^89^Sr, ^186^Re, and ^153^Sm are used in bone pain palliation, most of them have some limitations and disadvantages. For example, as a result of their destructive side effects, which originated from their high beta-energy particles (like ^32^P and ^89^Sr), some of these agents are not routinely used. Although ^186^Re labeled complexes are used in some countries at present, preparation of this radiopharmaceutical is not easily practicable, which burdens a kind of limitation to its use. However, with ^177^Lu, a β^-^emitter, delivering the appropriate dose to skeletal lesions with minimal bone marrow suppression can be achieved. Again, gamma photon emission with low abundance and sufficient energy from ^177^Lu allows for simultaneous scintigraphy studies and dosimetric evaluation.

In the present study, we intended to develop a ready-to-use kit for the preparation of ^177^Lu-EDTMP and evaluate the properties of the developed kit in view of the quality required for medicinal products for human use. According to this, first, the EDTMP ligand was synthesized, and its structure was determined using authentic spectroscopic methods. The results were in agreement with commercial reference samples.

Preparation of a radiopharmaceutical from a ready-to-use kit and injection into the patient without final purification needs kit optimization and quality check [17]. The radiolabeling optimization study was performed with two different salts of EDTMP based on a literature search [19]. The researchers used the natrium/calcium salt of the ligand in order to make the comparison to the commercially available kit of ^153^Sm-EDTMP. However, we found similar labeling yields for both salts of the ligand. The radiolabeling yield of EDTMP is pH-dependent, and the highest yield is obtained when the pH of the reaction mixture is between 6 and 9 [8, 20]. To obtain a final pH above 6.5, the pH of the ^177^LuCl_3_ solution used for the labeling was kept between 5.0-6.0.

Optimization was then followed by kit production. Lyophilization is the critical step in kit production. The water is removed from the product, resulting in a dry powder, which determines the shelf life of the product [17]. The >98% radiochemical purity of the ^177^Lu labeled product up to 48 hours proved the stability of the kit. The powder form of the EDTMP also enabled the ligand to be dissolved in the radioisotope solution (^177^LuCl_3_).

Quality checks of the reconstituted kit confirmed the sterility, absence of pyrogenicity, radiochemical, and radionuclidic purity of the final product. This shows that EDTMP could be labeled with ^177^Lu at 30 min and room temperature with a high radiochemical yield (>98%). The only impurity was the free ^177^Lu. Preparation of the ^177^Lu-EDTMP complex is very simple, and the complex is quite stable. Oxygen-containing phosphonic acid groups of the EDTMP can be complexed with the oxygen seeker Lu^+3^ ions [21].

## CONCLUSION

^177^Lu labelled radiopharmaceuticals can be prepared manually using automated modules. High investment costs to meet GMP requirements in these facilities are a major disadvantage. In contrast, the reconstitution of the ready-to-use kit with sterile radionuclide solution is a fast and repeatable process with minimal cost and expertise.

In the present study, we intended to develop a ready-to-use kit for the preparation of ^177^Lu-EDTMP and evaluate the properties of the developed kit in view of the quality required for medicinal products for human use. Our findings demonstrated that ^177^Lu-EDTMP could be prepared for routine use in nuclear medicine practices using the developed ready-to-use kit based on the optimized labeling procedure. Commercial manufacturing of ready-to-use kits will expand the use of 177Lu-EDTMP in routine nuclear medicine practices.

## Figures and Tables

**Fig. (1):**
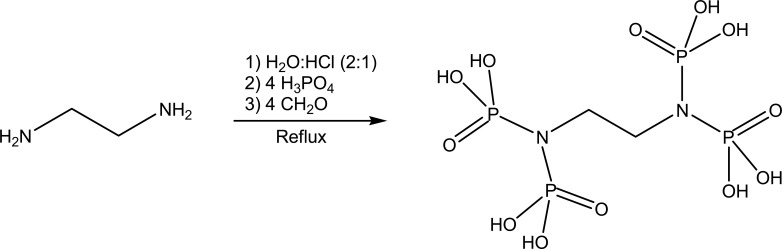
The synthesis scheme of ethylenediaminetetramethylene phosphonic acid (EDTMP).

**Table 1:** Labeling yields of two different salt forms of EDTMP with ^177^Lu.

**^177^Lu amount (mCi)**	**Salt form of EDTMP**	**Incubation (min)**	**Yield (%)**
100	Na-EDTMP	30	99.54
100	Na/Ca-EDTMP	30	99.41

**Table 2:** The radiochemical purity of 177Lu-EDTMP complex stored at 25 °C over the period of 48 hours.

**Time (hour)**	**Radiochemical Purity (%)**
0	99.46
6	99.43
12	99.32
24	99.10
48	99.00

## Data Availability

The data that support the findings of this study are available from the corresponding author upon request.
